# Identification and Expression Profiling of Protein Phosphatases (*PP2C)* Gene Family in *Gossypium* *hirsutum* L.

**DOI:** 10.3390/ijms20061395

**Published:** 2019-03-20

**Authors:** Hamna Shazadee, Nadeem Khan, Jingjing Wang, Chencan Wang, Jianguo Zeng, Zhongyi Huang, Xinyu Wang

**Affiliations:** 1 College of Life Science, Nanjing Agricultural University, Nanjing 210095, China; 2018116157@njau.edu.cn (H.S.); 2017116115@njau.edu.cn (J.W.); 2018116102@njau.edu.cn (C.W.); 2016116113@njau.edu.cn (J.Z.); 2018116101@njau.edu.cn (Z.H.); 2 State Key Laboratory of Crop Genetics and Germplasm Enhancement, Ministry of Science and Technology/College of Horticulture, Nanjing Agricultural University, Nanjing 210095, China; 2016104235@njau.edu.cn

**Keywords:** protein phosphatase (PP2C), cotton, syntenic relationships, expression patterns, evolutionary analysis

## Abstract

The protein phosphatase (*PP2C*) gene family, known to participate in cellular processes, is one of the momentous and conserved plant-specific gene families that regulate signal transduction in eukaryotic organisms. Recently, PP2Cs were identified in *Arabidopsis* and various other crop species, but analysis of PP2C in cotton is yet to be reported. In the current research, we found 87 (*Gossypium* *arboreum*), 147 (*Gossypium* *barbadense*), 181 (*Gossypium* *hirsutum*), and 99 (*Gossypium* *raimondii*) PP2C-encoding genes in total from the cotton genome. Herein, we provide a comprehensive analysis of the *PP2C* gene family in cotton, such as gene structure organization, gene duplications, expression profiling, chromosomal mapping, protein motif organization, and phylogenetic relationships of each species. Phylogenetic analysis further categorized *PP2C* genes into 12 subgroups based on conserved domain composition analysis. Moreover, we observed a strong signature of purifying selection among duplicated pairs (i.e., segmental and dispersed) of *Gossypium* *hirsutum*. We also observed the tissue-specific response of *GhPP2C* genes in organ and fiber development by comparing the RNA-sequence (RNA-seq) data reported on different organs. The qRT-PCR validation of 30 *GhPP2C* genes suggested their critical role in cotton by exposure to heat, cold, drought, and salt stress treatments. Hence, our findings provide an overview of the *PP2C* gene family in cotton based on various bioinformatic tools that demonstrated their critical role in organ and fiber development, and abiotic stress tolerance, thereby contributing to the genetic improvement of cotton for the resistant cultivar.

## 1. Introduction

The protein kinases (PKs) and protein phosphatases (PPs) are known to regulate the protein function, and are the fundamental molecular mechanism, by reversing protein phosphorylation during cellular signaling. Thus, it is involved in many biological processes, such as signal transduction, development, and environmental stimuli [1]. The PKs phosphorylate largely serine (Ser), threonine (Thr), and tyrosine (Tyr), whereas PPs can reverse this functioning by eliminating the phosphate group [2]. Mainly, the PPs are subcategorized into three major groups based on their requirement for substrate specificity, namely Ser/Thr phosphatases (STPs), protein Tyr phosphatases (PTPs), and dual-specificity phosphatases (DSPTPs) [3,4]. Moreover, based on crystalline structure, amino acid sequence and compassion to specific inhibitors (okadaic acid and cyclosporine-A), the PTPs are further classified into phosphoprotein metallophosphatase (PPM) and phosphoprotein phosphatases (PPP) [5]. The PPM family mainly involves Mn^2+^- or Mg^2+^-dependent protein phosphatase (PP2C) and pyruvate dehydrogenase phosphate, however, the PPP family includes different types of protein phosphatase, including PP1, PP4, PP5, PP6, PP7, PP2A, and PP2B [5]. In the PPP family, PP2A rigorously affects root hair growth during the elongation phase by denaturing the shape of the cells [6].

PP2C are evolutionarily conserved from Archaea to higher plants that pointedly modulate stress signaling pathways and reverse the stress-induced PK cascades to complex environmental stimuli [3]. From various literature, several key stress-responsive protein kinases genes have been extensively studied and proven to respond in diverse stress conditions including biotic and abiotic factors [7,8]. In *Arabidopsis*, several members of PP2c such as PLL4 and PLL5 (POL-like gene) are known to adjust leaf development, though with no obvious functions within the meristem [9]. 

In *Arabidopsis* and rice, 112 and 132, respectively, candidate *PP2C* genes have been characterized into various groups [10,11]. In particular, group A of *Arabidopsis* PP2Cs (e.g., ABI1 and ABI2), is accompanied by abscisic acid (ABA) signaling, group B is known to stimulate mitogen-activated protein kinase (MAPK) signaling, and group C is involved in the regulation of flower development [12]. Moreover, several members of PP2C have been implicated as a negative regulator in mediated stress signaling within ABA [13,14,15]. In higher plants, PP2Cs act as negative regulators of the ABA signaling pathway and reduce tolerance against oxidative stress [16]. Proteins encoded by these candidate genes play a critical role in various abiotic stress signaling such as salt, drought, and freezing [17,18,19,20]. Crop productivity encounters a number of abiotic stress signaling including salt, drought, heat, and osmotic stress in the environment. Thus, plants have developed complex molecular mechanisms to implement and survive during adverse growth conditions. Intriguingly, it can be contingent on quite a few factors, like adaptive alterations in their structure, physiology, and gene expression of some regulatory proteins [16]. In reaction to drought stress, the interaction of PP2C i.e., MPK3 and MPK6, assists plants in stomatal assimilation to thwart water loss [21], such as in the *Arabidopsis* [22]. Taken together, the above studies have established the diverse role of *PP2C* genes in plant development and environmental stresses. Hence, it is indispensable to probe into the identification and functional description of the *PP2C* gene family, which will cement the base for understanding its essential molecular mechanism in stress signaling.

Cotton, as an oil crop and an important source of natural textile fiber, plays a crucial role in agriculture and industry all around the world. However, its production is mainly constrained due to various abiotic and biotic stress conditions. The release of *Gossypium* whole-genome data in four different cotton species, such as *Gossypium arboreum* [2], *Gossypium barbadense* [23], *Gossypium hirsutum* [24], and *Gossypium raimondii* [25] and their publicly available database allows us to comprehensively characterize the *PP2C* gene family based on bioinformatic tools. We further compared the *PP2C* genes between *Gossypium* and *Arabidopsis*, to explore and identify both shared and specific subgroups. Following the gene structure organization analysis, conserved protein motifs, and cis-elements, we traced the duplication gene pairs and their evolutionary divergence that likely resulted in the widespread extension of the *PP2C* gene family. In order to shed light on some critical *PP2C* genes, their associated indigenous functional roles were further exposed to heat, cold, drought, and salt stress conditions. Additionally, the transcriptional profiling of the *PP2C* genes for various tissues and fiber development and qRT-PCR analysis of 30 genes were analyzed and compared. Therefore, to our knowledge, this is the first systematic report of the genome-wide expression dynamics of the *PP2C* genes in cotton species, and it is necessary to carry out a comprehensive study to understand the regulation of phosphatases in cotton during stress and development.

## 2. Results

### 2.1. Characterization of PP2C Gene Family in Cotton

The *Arabidopsis* 94 *PP2C* genes were obtained from TAIR (http://www.arabidopsis.org) with the help of Interpro scan domain (IPR001932) and then used as queries against *G. arboreum*, *G. barbadense*, *G. hirsutum*, and *G. raimondii* databases. This search resulted in 87, 147, 181, and 99 *PP2C* genes in *Gossypium* species i.e., *G. arboreum, G. barbadense, G. hirsutum,* and *G. Raimondii,* respectively. In the current study, we used the following nomenclature system for the *PP2C* genes to distinguish each PP2C from the homology of *Arabidopsis* and *Gossypium* species: GaPP2C (GaPP2C1–GaPP2C87) *G. arboreum*, GbPP2C (GbPP2C1–GbPP2C147) *G. barbadense*, GhPP2C (GhPP2C1–GhPP2C181) *G. hirsutum*, and GrPP2C (GrPP2C1–GrPP2C99) *G. raimondii*. We have studied different gene features of the *PP2C* genes including the chromosomal location, coding sequence length (CDS), protein length (aa), molecular weight (MW), isoelectric point (PI), grand average of hydropathicity (GRAVY), aliphatic index, and subcellular localization (Supplementary Tables S1–S4). In general, most of the PP2C proteins were in the range of 254–899 (GaPP2C1–GaPP2C66), 240–1630 (GbPP2C3–GbPP2C27), 263–794 (GhPP2C38–GhPP2C135), and 264–1101 (GrPP2C27–GrPP2C67) amino acids. The MWs of the proteins ranged from 27.99–100.38 (GaPP2C1–GaPP2C66), 26.45–181.58 (GbPP2C38–GbPP2C27), 28.65–100.47 (GhPP2C38–GhPP2C133), and 28.83–122.57 (GrPP2C27–GrPP2C1), the PIs ranged from 4.79–9.18 (GaPP2C28–GaPP2C7), 4.41–9.27 (GbPP2C96–GbPP2C135), 4.53–9.27 (GhPP2C57–GhPP2C177), and 4.64–9.27 (GrPP2C68–GrPP2C86), and the GRAVY ranged from −0.59–0.04 (GaPP2C66–GaPP2C64), −0.70–0.06 (GbPP2C27–GbPP2C102), −0.591–0.056 (GhPP2C132–GhPP2C127) and −0.587–0.071 (GrPP2C78–GrPP2C71). In our study, most of the *PP2C* genes showed hydrophilic properties with negative values, only a few of them were hydrophobic in nature with a positive value. The predicted subcellular localization results displayed that most of the PP2C proteins were confined in different organelles such as the chloroplast, the nuclear region, mitochondria, cytoplasm, and others (Supplementary Tables S1–S4).

### 2.2. Phylogenetic and Genomic Distribution and Organization Analysis of PP2C Gene Family

**Figure 1:**
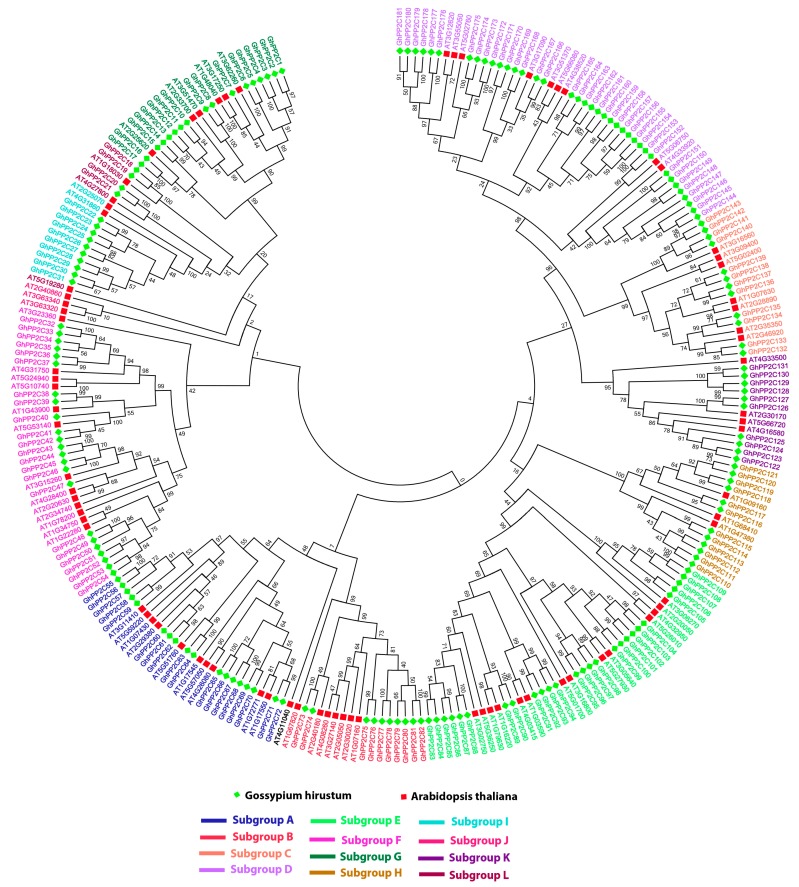
Phylogenetic relationship of *PP2C* genes between *G*. *hirsutum* and *A. thaliana*. The phylogenetic tree was constructed by MEGA 7 using the Maximum Likelihood Method (1000 bootstrap). Genes of protein phosphatase (PP2C) from different subgroups are marked with various color.

**Figure 2:**
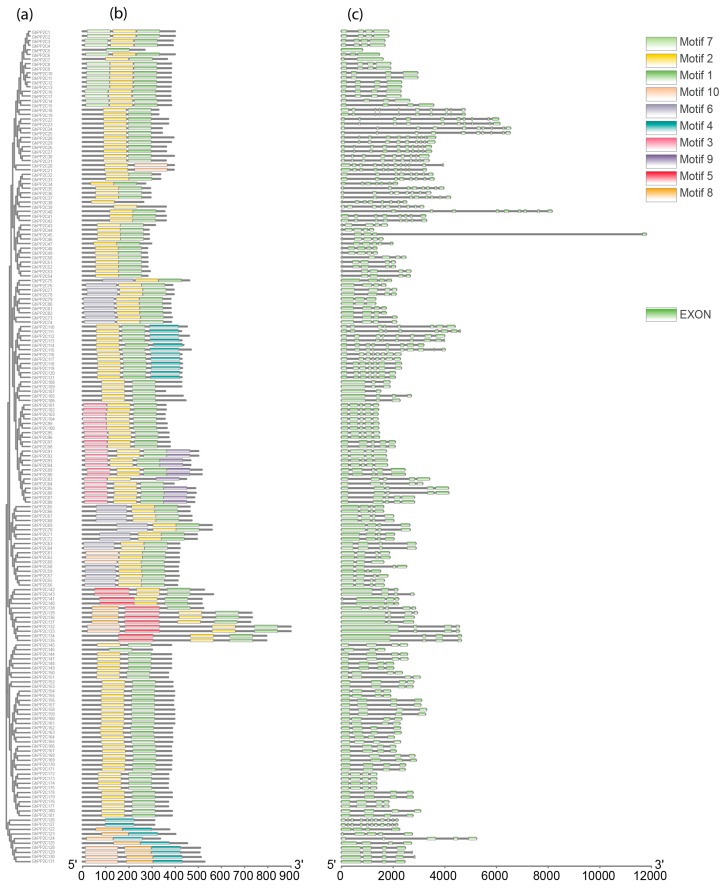
(**a**) (**b**) and (**c**). Phylogenetic relationships, gene structure, the exon-intron, and upstream/downstream region are represented, respectively. The phylogenetic tree was constructed by MEGA 7 using the Maximum Likelihood Method (1000 bootstrap). At the bottom of the figure, the relative position is proportionally displayed based on the kilobase scale.

To study the phylogenetic relationship of the *PP2C* genes among cotton plants (*G. arboreum*, *G. barbadense*, *G. hirsutum*, and *G. raimondii*), a maximum likelihood (ML) tree was constructed with *Arabidopsis* using MEGA 7.0. The phylogenetic tree revealed that the *PP2C* genes can be further divided into 12 subgroups, as previously reported [26,27] (Supplementary Figures S1–S3). Moreover, 181 *GhPP2C* genes were clustered into 12 subgroups (A–L) with *Arabidopsis*. In the phylogenetic tree, subgroup D had the most members of the *PP2C* genes (38), followed by subgroup E (27), and the least number of *PP2C* genes was observed in subgroup L, having 4 *GhPP2C* genes (Figure 1 and Figure 2a). Moreover, we also analyzed the conserved motifs and gene structure based on phylogenetic relationships to provide insight into the structural features of the PP2C members in *G. hirsutum*. For GhPP2C proteins, 10 conserved motifs were acquired with the help of MEME. The majority of the PP2C family contained motifs 7 and 2, except a few genes showed motifs 3 and 4. This indicates that all the identified *PP2C* genes have typical family features and the proteins classified into the same subgroup share similar amino acid sequences (Figure 2b). Alongside, their logos were obtained by online MEME server. A total of 10 types of consensus motifs were obtained in all of the GhPP2C proteins and their distribution patterns are presented in Supplementary Figure S4. To better understand the gene structure of *PP2C* genes in cotton, exon-intron organizations of these genes were also tested (Figure 2c) [26], and most of the subgroups contained 1-10 introns. These results specify that *PP2C* genes in the same subgroup show more or less similar exon-intron organization.

### 2.3. Chromosomal Localization and Syntenic Relationships of PP2C Gene Family

**Figure 3:**
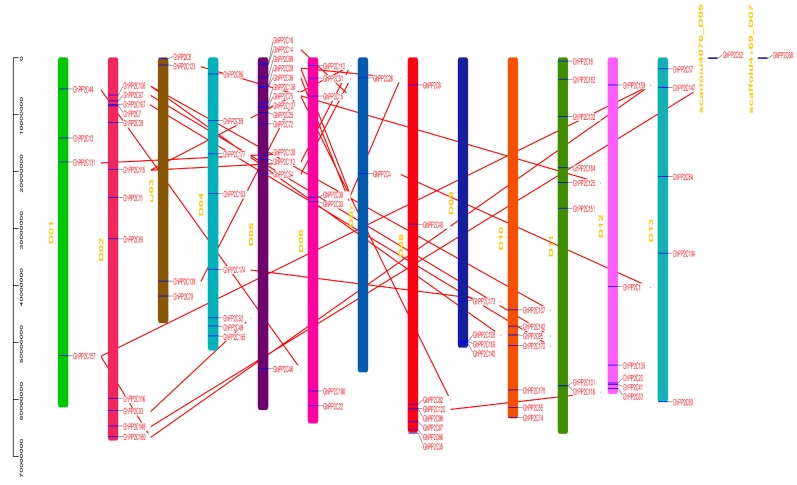
Chromosomal locations of the PP2C D genome of cotton­ were obtained from the Generic File Format (GFF) file and displayed using TBtools software. The red line indicates the collinear relationship for different chromosomes.

**Figure 4:**
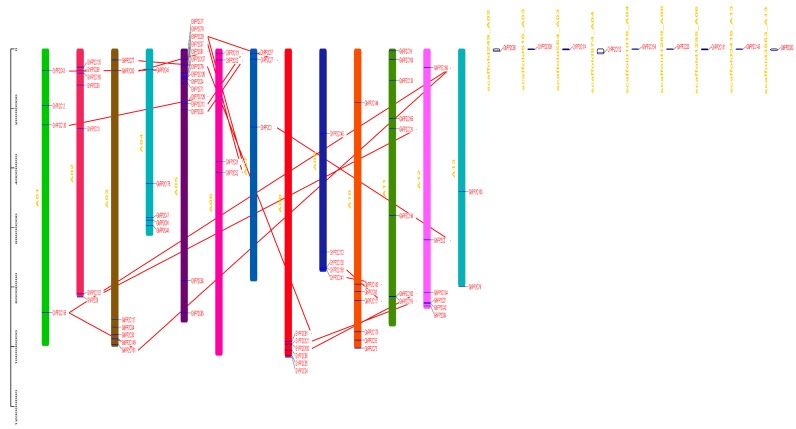
Chromosomal locations of PP2C A genome of cotton­ were obtained from the GFF file and displayed using TBtools software. The red line indicates the collinear relationship for different chromosomes.

The chromosomal localization of A and D genomes of *G. hirsutum* was analyzed using Tbtools software. A total of 89 *GhPP2C* genes were allocated in D genome (D01–D13) ranging from 2–14 genes per chromosome and only 2 genes were found on the scaffold. Moreover, every chromosome showed variation in a number of genes, such as D04 exhibited the highest number of *GhPP2C* genes (14), followed by 12 genes in D02, and the least number of genes (2) were recorded for D07 (Figure 3). On the other hand, we also demonstrated the chromosomal localization for A genome (A01–A12). A total of 79 genes were found ranging from 2–15 per chromosomes and 9 of them were located on the scaffold. The highest number of genes was found on A05 (15), followed by A11 (8), and the least number of *PP2C* genes (2) were found on A13 (Figure 4). These findings suggested that GhPP2C were allocated unevenly to different chromosomal locations. 

**Figure 5:**
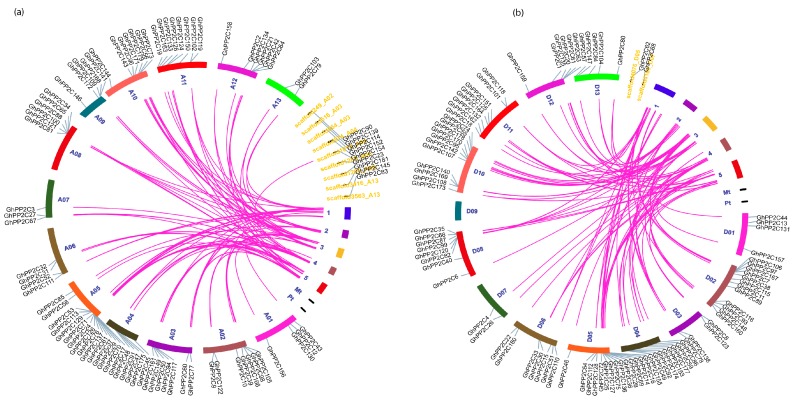
(**a**) and (**b**). Chromosomal locations of PP2C (A and D) genome of cotton­ were obtained from the GFF file and displayed using TB tools software. The pink line indicated the collinear relationship for different chromosomes.

Moreover, a collinear correlation was also demonstrated between *G. hirsutum* and *Arabidopsis* (A and D genomes) (Figure 5a,b). To validate our results, we also performed a collinear relationship of *PP2C* genes using only *G. hirsutum* (Supplementary Figure S5). The results exhibited high conservation among PP2C members between the A and D genomes of cotton.

### 2.4. Analysis of Putative Regulatory Cis-Element and Gene Duplication Analysis of PP2C Gene Family in Cotton

**Figure 6:**
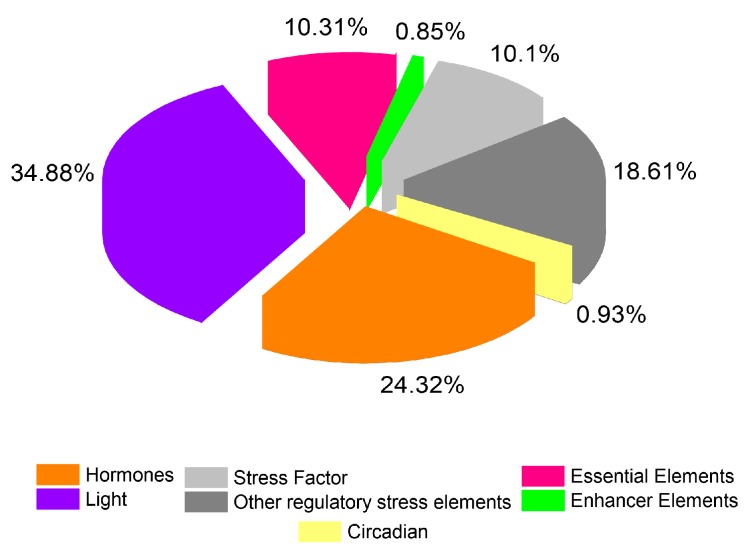
The ratio of various cis-elements in cotton.

In the promoter region of *PP2C* genes, cis-acting elements play a critical role as stress-adaptive signaling in plants by interacting with their cognate transcription factor (TF). For instance, abscisic acid (ABA)-responsive elements (ABREs) are involved in salt, drought, and ABA signaling [28]. LTR is crucial to chilling response and regulation [29]. Likewise, TCA-elements and TGACG-motif are responsive to salicylic acid (SA) and MeJA treatments [30]. The exploration of the cis-acting element of *G. hirsutum PP2C* genes was executed by using the PlantCARE database and seven common cis-regulatory elements were briefly summarized (Figure 6 and Supplementary Table S5). The results unveiled that most of the genes contributed in various signaling pathways such as phytohormones and biotic and abiotic regulatory stress factors. On the other hand, *PP2C* genes are recognized to show a major part in both biotic and abiotic stress phenomena. Most of the genes were highly (34.88%) responsive to light (AE-BOX, BOX-4, LAMP-ELEMENTS, GAG-motif, GATA-motif), followed by (24.32%) hormones (ABRE, CGTCA, TGA, TCA, AuxRe, GARE-motif), (18.61%) and other regulatory cis-elements (HD-ZIP3, o2-site, AT-Rich elements, CAT-BOX, A-Box, EIRE), while the fewest genes (0.93% and 0.85%) were mainly counted in circadian and enhancer elements (5UTR Py-rich stretch, TA-Rich Region, and GC-motif), respectively. In plants, the circadian cis-regulatory element is known to control the circadian rhythms. Moreover, these results indicate that *PP2C* genes are vital in various biotic-abiotic/hormone signaling which might be hypothesized by their diversity in nature.

**Figure 7:**
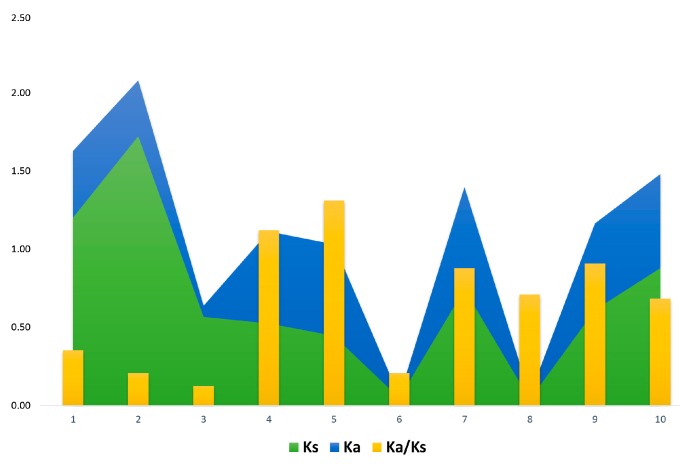
The correlation between *Ks* and *Ka* for duplicated genes (segmental and dispersed).

For gene duplication analysis, we used MCScanX to determine the types of gene duplications and the results suggested most of the genes were segmental (167) and few of them were dispersed (12); thus, indicating that segmental duplication plays a major contribution to the expansion of the *PP2C* gene family. Moreover, the *PP2C* genes might have experienced functional discrepancy due to gene duplications, and few of them might have lost their unique functions, developed novel functions, or preserved partition of innovative functions [31,32]. During the evolutionary processes, genes are often exposed to various selective pressures such as positive, neutral, and purifying selection. Additionally, for a better understanding of the selection pressure between the duplicated genes, we calculated the *Ka/Ks* ratios among selected genes from segmental and dispersed (Figure 7 and Table 1). It was shown that only two pairs have a positive selection (>1) *Ka/Ks*, while the rest were purifying in nature with <1.00, reducing divergence after duplication.

### 2.5. Expression Profiling of PP2C Gene Family in Different Tissues of G. hirsutum

**Figure 8:**
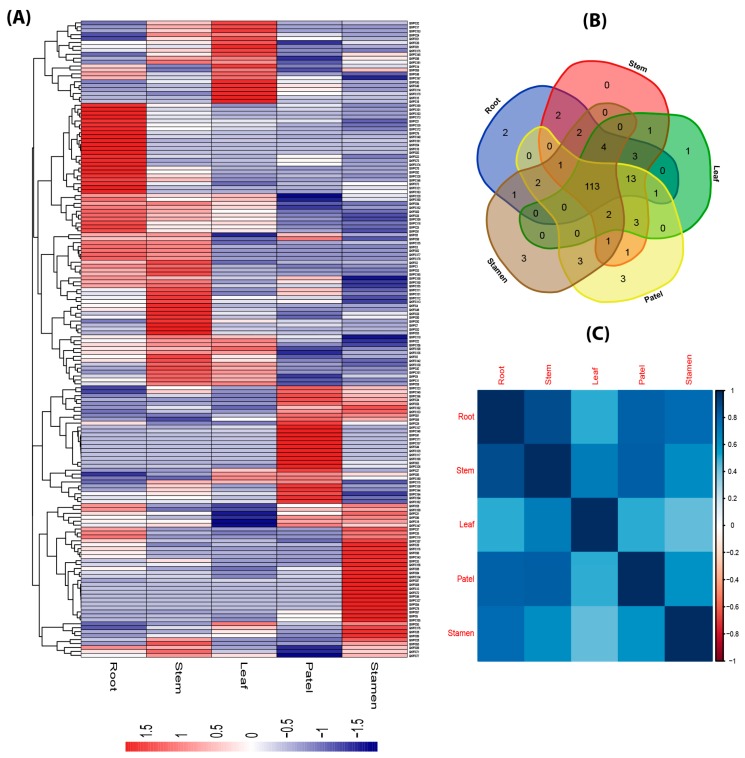
(**A**) Heat map of expression profiles (in log_2_-based fragments per kilobase of transcript per million fragments mapped (FPKM)) for PP2C in the five various tissues—root, stem, leaf, patel, and stamen. The expression levels are indicated by the color bar. (**B**) Venn diagram analysis of the tissue expression of PP2C. (**C**) Pearson’s correlation coefficients (PCCs) of FPKM-based values of 181 GhPP2Cs in different tissues using Rstudio.

To gain insight into the tissue-specific expression patterns of the cotton, previously reported [24] transcriptome data was utilized for various tissues (root, stem, leaf, patel, and stamen) and fiber development (3, 6, 9, 12, and 15 days post anthesis). For instance, Figure 8a reveals the expression levels of *PP2C* genes drastically varied in various tissues and most numbers of them were highly expressed. However, the expression of a few genes (GhPP2C12, 16, 46, 45, 47, 50, 57, 74, 79, 95, 97, 122, 124, 125, 132, 141, 154, 159, and GhPP2C170) did not show any striking expression in any tissues. Though, some of the *PP2C* genes were expressed in one or more tissues (GhPP2C15, 17, 18, 27, 40, 44, 46, 64, 68, 72, 78, 117, 127, 129, 138, 151, 168, and GhPP2C171). Intriguingly, the tissue-specific clustering (Figure 8b) showed that root (2), stamen (3), patel (3), and leaf (1) genes were commonly involved in the tissue developmental role in cotton. To gain further insights into the connection between these *GhPP2C* genes in tissue-specific responses, a correlation analysis was established based on the Pearson correlation coefficients (PCCs) (*p* = 0.05). Results showed a higher positive correlation among various specific tissues (Figure 8c and Supplementary Table S6). 

**Figure 9:**
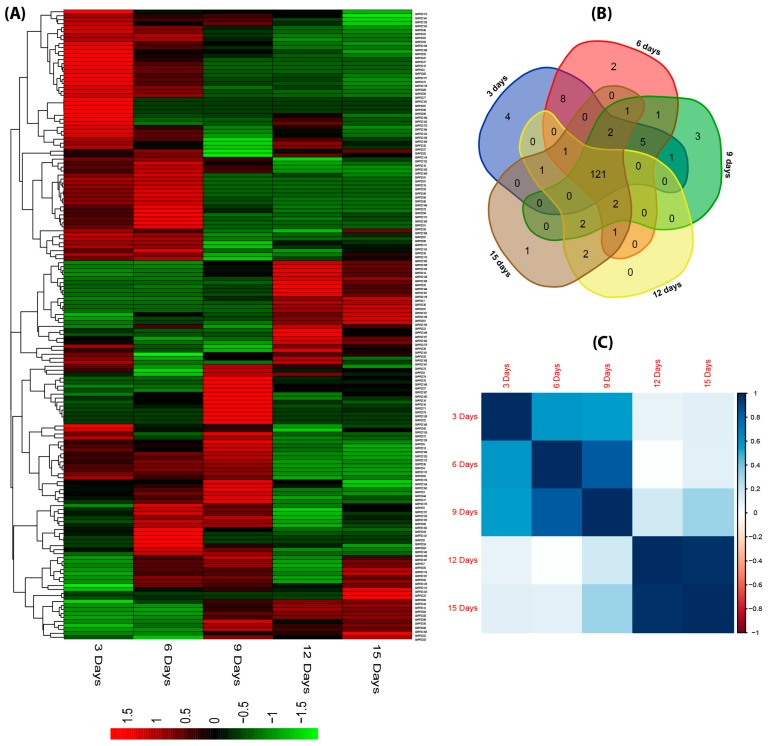
(**A**) Heat map of expression profiles (in log_2_-based FPKM) for PP2C in the five fiber developments: 3 days post anthesis (DPA), 6, 9, 12, and 15 DPA. The expression levels are indicated by the color bar. (**B**) Venn diagram analysis of the fiber development of PP2C. (**C**) Pearson’s correlation coefficients (PCCs) of FPKM-based values of 181 GhPP2Cs in different fiber development using Rstudio.

Furthermore, the expression patterns of fiber developmental stages (3, 6, 9, 12, and 15 days post anthesis) exhibited a dynamic expression level. Majority of the *PP2C* genes were highly expressed (Figure 9a), but some of them were not expressed, such as GhPP2C11, 21, 25, 26, 31, 40, 42, 45, 54, 76, 80, 86, 89, 91, 98, 100, 110, 117, 137, 148, 155, 171, and GhPP2C178, respectively. As shown in Figure 9b, the clustering analysis indicated the common developmental genes in 3 days post anthesis (DPA) (4), 6DPA (2), 9DPA (3), and 15DPA (1), respectively. The PCCs-based correlation analysis of the relative gene expression of selected genes suggested a high positive correlation and low inverse correlation between selected genes. In addition, some genes exhibited an inverse correlation among various fiber developmental stages (Figure 9c and Supplementary Table S6). These findings suggested that *PP2C* genes share diverse and high expression patterns in tissues and fiber development, which implied *PP2C* genes are conserved. 

### 2.6. qRT-PCR Analysis of the Candidate PP2C Gene Family in Response to Various Stresses

**Figure 10:**
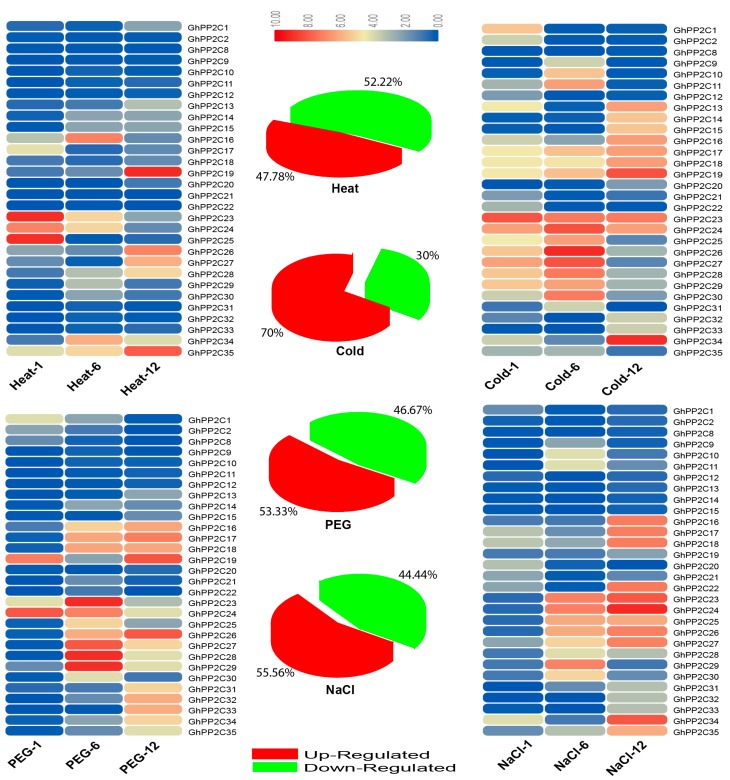
Relative expression analysis by qRT-PCR of *PP2C* genes under heat, cold, drought, and salt stressing in *G*. *hirsutum*.

**Figure 11:**
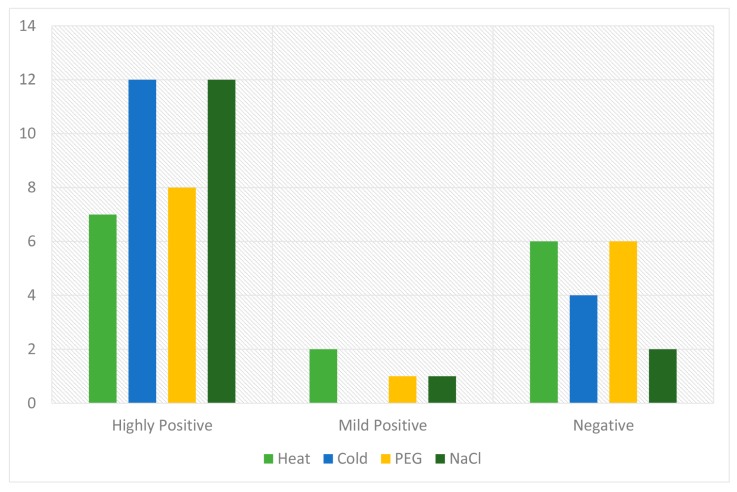
The Pearson’s correlation coefficient (PCC) values under response to heat, cold, drought, and salt treatments.

A prediction of the cis-regulatory elements indicated that *GhPP2C* genes may participate in responses to heat, cold, drought, and NaCl stress tolerance. Moreover, the expression profiling of PP2C has been studied in various species after exposure to abiotic–biotic and hormones stresses [3,26]. To verify this hypothesis, we subjected the cotton seedlings to four various abiotic stress treatments such as heat, cold, drought, and NaCl stress, and then carefully selected 30 genes for qRT-PCR. The results showed that some *GhPP2C* genes exhibited high transcript levels after exposure to multiple treatments, but a few of them were induced by one or more treatments. For instance, heat stress possesses the dominant portion of down-regulated genes (52%). However, cold stress showed 70% of the genes were up-regulated and 30% decreased in transcript level, followed by drought stress, which exhibited about 53% and 47% of up- and down-regulated *GhPP2C* genes, respectively. On the other hand, exposure to salt stress resulted in 56% up-regulation and 44% down-regulation in genes (Figure 10). Among these 30 genes, we also calculated the Pearson correlation coefficient (PCC) based on the expression by making three categories (i.e., highly positive >0.5, mild positive <0.5 and >0, and negative correlation <0). The results emphasized that both cold and salt stress showed 12 each PCC values having a highly positive correlation, while negative correlation was recorded in heat and drought with 6 each PCC values (Figure 11 and Supplementary Table S6). Taken together, all the 30 genes were induced by different abiotic stresses, but the diversity in the expression profiling of *GhPP2C* genes may suggest that these genes may be critical to abiotic-stress responses.

## 3. Discussion

In this study, we systematically investigate the *PP2C* genes based on the genome-wide analysis. Although the *PP2C* gene family has been in many species, the knowledge of *PP2C* genes in cotton is yet to be elucidated and their systematic analysis has not been reported. Previously, the *PP2C* gene family has been reported in different plant species such as maize [12], rice [11], *Arabidopsis* [10], hot pepper [33], banana [34], and *Brachypodium distachyon* [3]. Thus, we observed high variations in the number of *PP2C* genes that might be present during whole-genome duplication (WGD) events. For exploring new biological functions, evolutionary implications and its expansions are mainly based on gene duplications [35]. Therefore, to study evolutionary analysis and polyploid formation, cotton is an excellent model and ideal crop by being typically allotetraploid [36]. The importance of evolutionary analysis is further reflected in the fact that most of the angiosperms have undergone either one or multiple polyploidization events [37,38,39]. Herein, we systematically found PP2C-encoding genes in four cotton species—87 (*Gossypium arboreum*), 147 (*Gossypium barbadense*), 181 (*Gossypium hirsutum*), and 99 (*Gossypium raimondii*). Cotton species represent the high number of genes, specifying that the *PP2C* gene family have undergone extensive expansion during the evolution of cotton. In the current study, a comprehensive genome-wide analysis was executed, such as gene identification, gene structure organization, phylogenetic characterization, syntenic relationships, chromosomal localization, and gene duplications. Moreover, transcriptional profiling of 30 *PP2C* genes was also performed, after exposure to heat, cold, drought, and salt stress was also carried out [12,27]. 

In this study, the subcellular predictions for most of the members of GaPP2C, GbPP2C, GhPP2C, and GrPP2C were mainly located in different organelles such as the chloroplast, nuclear region, mitochondria, cytoplasm, and others. In addition, the gene characteristics of GaPP2C, GbPP2C, GhPP2C, and GrPP2C drastically vary including the protein length (aa), molecular weight (MW), predicted isoelectric point (PI), and a grand average of hydropathicity (GRAVY), signifying that different PP2C proteins may play complex roles in variable microenvironments.

The estimate of evolutionary patterns, such as the rate of computing the selection pressure analysis (*Ka/Ks*), provides useful information about purifying, positive, and neutral selections of gene pairs during the rate of divergence [40]. In evolutionary events, if the value of the *Ka/Ks* ratio is <1.00, this represents purifying selection, a *Ka/Ks* ratio of 1.00 indicates neutral selection, and/or *Ka/Ks* >1.00 depicts positive selection [41,42]. Similarly, during evolutionary processes and expansion of a gene family, these indicators are used for the selection history. In this study, we calculated these values among segmental and dispersed pairs of *PP2C* genes with the help of the MEGA7.0 program. Thus, we estimated the selection pressure analysis of duplicated genes (i.e., segmental and dispersed) pairs. The majority of the GhPP2C pairs showed *Ka/Ks* ratios of less than 1.00, indicating the purifying selection, and only two pairs showed values more than 1.00, speculating the positive selection [31].

Plant productivity is always uncertain due to a range of climatic challenges such as heat, cold, drought, and salt stress being the major factors in limiting crop production. Previous studies reported that *AtPP2CG1* regulates positively against salt tolerance in *Arabidopsis* and is induced by drought, salt, or exogenous ABA treatment [43]. In *Arabidopsis*, two members of *PP2C* genes responded inversely; for example, *AP2C1* expression was powerfully tempted by drought, wounding, and cold but *AP2C2* was slightly prompted by these treatments too [44]. These findings highlighted the significance of critical members of the *PP2C* gene family in the model plant *Arabidopsis*, but their specific functions may be significantly varied in cotton. For achieving gene expression patterns in diverse growth phases of GhPP2C, we systematically analyzed the previously reported transcriptome data in various tissues (root, stem, leaf, patel, and stamen) and fiber development (3, 6, 9, 12, and 15 DPA). These results showed that most of the PP2C were highly varied, speculating the high diversity in their functions. However, few genes have shown tissue-specific expression, indicating their common importance to plant development. In *GhPP2C* genes, we also explored the promoter regions for identification of common conserved cis-regulatory elements. As a consequence, these results exhibit the participation of *PP2C* genes in various hormone signaling, including both biotic and abiotic stress factors. In the present study, to provide validation to our hypothesis, we also tested 30 candidate genes under various stresses using qRT-PCR. The majority of *PP2C* genes showed high striking transcriptional changes by exposure to heat, cold, drought, and salt stress, suggesting that *PP2C* genes might be crucial to stress tolerance in cotton. Noticeably, recent studies in *Arabidopsis* and rice also uncovered the pivotal role of PP2C candidate genes [11,45], however, in cotton, it is largely obscured. Moreover, 30 candidate genes involved in expression profiling demonstrated their critical role in *Gossypium hirsutum*. 

Therefore, taken together, the results of our study provided valuable insight, signifying that PP2C has provided a stepping stone to the molecular mechanism in cotton. Alongside, the differential expression profiling and gene duplications analysis might have experienced functional divergence, and their further study will help considerably in improving the crop yield and quality and cultivating new resistant varieties.

## 4. Conclusions

We analyzed the genome analysis, evolutionary rates, and molecular characterization of *PP2C* genes in the *Gossypium* genome. Herein, we identified 87 (*Gossypium arboreum*), 147 (*Gossypium barbadense*), 181 (*Gossypium hirsutum*), and 99 (*Gossypium raimondii*) *PP2C* genes by bioinformatics analysis in cotton species. Gene synteny analysis showed that GhPP2C are highly conserved, while the gene duplications analysis reflected that only segmental duplication plays a starring role in the *PP2C* gene extension in cotton. Also, the results of the phylogenetic analysis categorized the *PP2C* genes into 12 subgroups. We further explored the previously published RNA-sequence (RNA-seq) data to compare the tissue-specific response of *PP2C* genes and their critical role in organ development and fiber. Various *PP2C* genes responded promptly to abiotic stresses, including heat, cold, salt, and drought, suggesting their crucial role in abiotic stress tolerance. As a consequence, our findings will facilitate advanced research on the functional analysis of *PP2C* genes regarding their critical role in tissues, fiber development, and abiotic stress tolerance.

## 5. Materials and Methods 

### 5.1. Data Resources for Sequence Retrieval

For identification of *PP2C* genes in *Gossypium* and other species, we utilized the Plaza 4.0 database (https://bioinformatics.psb.ugent.be/plaza/) with the help of InterPro PP2C domain “IPR001932”. The cotton genome sequences were downloaded from (https://www.cottongen.org/), and *A. thaliana* sequences were retrieved from TAIR (http://www.arabidopsis.org/). The domains of obtained GhPP2C proteins were further verified using the NCBI-Conserved Domain database (https://www.ncbi.nlm.nih.gov/Structure/cdd/wrpsb.cgi) search program and SMART databases (http://smart.embl-heidelberg.de/) [46]. Those proteins which lack PP2C domains were removed from further analysis. In addition, protein sequences that were found with obvious errors in their gene length or having less than 100 lengths were eliminated.

### 5.2. Multiple Sequence Alignment and Phylogenetic Analysis

The amino acid sequences of the GhPP2C proteins were used for further investigation, and multiple sequence alignment was performed by MUSCLE [47] using MEGA 7 software with the default options [48]. The phylogenetic trees were constructed using the maximum likelihood (ML) method. In order to determine the reliability of the resulting tree, bootstrap values of 1000 replications were performed with the Jones, Taylor, and Thornton amino acid substitution model (JTT model), while keeping the other parameters as a default.

### 5.3. Calculation of the Ka/Ks for Duplicated Genes

The *Ka/Ks* ratios were calculated for duplicated (segmental and dispersed) gene pairs using MEGA 7.0 [48]. 

### 5.4. Conserved Motifs, Exon-Intron Structure Analysis, and Physicochemical Parameters of PP2C Proteins 

Conserved motif scanning of GhPP2C proteins was carried out through local MEME Suite Version 5.0.3. For this purpose, parameter settings were calibrated as follows: Maximum number of motifs 10, with a minimum width of 100 and a maximum of 150. The other parameters were set as default [49]. For the exon-intron structure, we used the Gene Structure Display Server (GSDS 2.0) (http://gsds.cbi.pku.edu.cn) [50]. The physicochemical properties of the proteins, including molecular weight (MW), isoelectronic points (pI), aliphatic index, and GRAVY values for each gene, were calculated using the ExPASY PROTPARAM tools (http://web.expasy.org/protparam/). The subcellular localization was predicted using the WOLF PSORT (https://wolfpsort.hgc.jp/) website.

### 5.5. Cis-Elements Predictions of GhPP2C

Every GhPP2C promoter sequence (selected as 2000 upstream bp) was imported in Generic File Format (GFF) file from the cotton genome. Then, the PlantCARE database (http://bioinformatics.psb.ugent.be/webtools/plantcare/html/) [51] was utilized to identify the cis-regulatory elements for promoters of each gene. 

### 5.6. Chromosomal Location and Synteny Correlation Analysis

The chromosomal location of *PP2C* genes was illustrated from top to bottom concerning their position in the genome annotation by using TBtools software [52]. For synteny gene analysis, the relationships were verified between the homologs of A. *thaliana* and *Gossypium hirsutum*. Circos (using TBtools software) program was applied to exhibit the syntenic relationships among the chromosomes of *G. hirsutum* and *A. thaliana* [52]. 

### 5.7. Pearson Correlation Analyses (PCC)

Pearson correlation analysis was performed with the help of Excel 2013 in order to evaluate the PCC values that were used for qRT-PCR according to a previously reported study [53]. As well, the PCC of fragments per kilobase of transcript per million fragments mapped (FPKM) values was implemented using RStudio (R program) at 0.05 (*p*-value) significance level. 

### 5.8. Plant Material and Treatments

In the present study, the germinated seeds of *G. hirsutum* cv. Junmian 1 were grown in plastic pots containing a mixture of soil and vermiculite (3:1). The pots were then placed in an artificial growth chamber for five weeks. The growth conditions were as follows: The temperature was set to 24/16 ºC, the photoperiod was 16/8 h, and the relative humidity was 65%–70%. The two-week-old seedling was exposed to specific treatment, as follows: For heat and cold treatments, seedlings were exposed to 38 ºC and 4 ºC, respectively. For NaCl and drought stress treatments, the seedlings were cultured in a nutrient solution medium with 250 µM and 6000 PEG (*w*/*v*). All treatments were carried out in continuous time intervals of 1, 6, and 12 h, respectively. Additionally, for every specific treatment, five randomly chosen whole seedlings were pooled to form a biological replicate. After that leaf samples were quick-frozen in liquid nitrogen and stored at −80 °C for further use. 

### 5.9. RNA Isolation and Transcriptional Profiling of GhPP2C under Various Stresses

Total RNA was isolated from the treated frozen leaves with Trizol (Invitrogen) following the manufacturer’s instructions. RNA was reverse-transcribed into cDNA using the Primer Script RT reagent kit (TAKARA, Dalian China) according to their instructions. Specific primers were designed using Becan Designer 7.9 and are presented in Supplementary Table S7. In order to check the specificity of the primers, we used the BLAST tool against the *Gossypium hirsutum* genome for confirmation. RT-PCR was performed according to the guidelines of previous studies [54]. Relative fold expression was calculated with the comparative Ct-method. The expression patterns of all *GhPP2C* genes were analyzed based on a previous study [55,56]. The cotton histone3 (AF024716) gene was used as the reference gene for qRT- PCR. In brief, the real-time PCR amplification reactions were performed on an ABI 7500 Real-Time PCR System (Applied Biosystems, California, CA, USA) using SYBR Green (Vazyme, Nanjing, China) with three replicates. The amplification parameters were denaturation at 95 °C for 10 min, 40 cycles of denaturation at 95 °C for 15 s, annealing at 60 °C for 15 s, and extension at 72 °C for 15 s.

High-throughput RNA-sequencing data [24] was utilized for various vegetative (root, stem, and leaf), floral (stamen and patel), and fiber tissues (3, 6, 9, 12, and 15 days post anthesis), respectively. Furthermore, gene expression levels were quantified by FPKM (fragments per kilobase of transcript per million fragments mapped) values, and heat maps were generated using an online omicshares tool (http://www.omicshare.com/) and TBtools software [52].

## Figures and Tables

**Table 1:** The gene with outlier *Ka/Ks* values and types of duplications i.e., segmental and dispersed genes.

Gene 1	Gene 2	*Ks*	*Ka*	*Ka/Ks*	Selection Pressure	Gene Duplications
*GhPP2C9*	*GhPP2C3*	1.20	0.43	0.354114713	Purifying Selection	Segmental
*GhPP2C10*	*GhPP2C6*	1.72	0.36	0.207076566	Purifying Selection	Segmental
*GhPP2C4*	*GhPP2C1*	0.57	0.07	0.125220459	Purifying Selection	Segmental
*GhPP2C7*	*GhPP2C8*	0.53	0.59	1.121904762	Positive Selection	Segmental
*GhPP2C11*	*GhPP2C2*	0.45	0.59	1.311659193	Positive Selection	Segmental
*GhPP2C89*	*GhPP2C90*	0.05	0.01	0.208333333	Purifying Selection	Dispersed
*GhPP2C114*	*GhPP2C146*	0.74	0.65	0.879032258	Purifying Selection	Dispersed
*GhPP2C83*	*GhPP2C84*	0.04	0.03	0.710526316	Purifying Selection	Dispersed
*GhPP2C139*	*GhPP2C180*	0.61	0.56	0.908346972	Purifying Selection	Dispersed
*GhPP2C62*	*GhPP2C23*	0.88	0.60	0.683731513	Purifying Selection	Dispersed

## Supplementary Materials

The following are available online at https://www.mdpi.com/1422-0067/20/6/1395/s1.
